# The Role of Gut Microbiome in Obesity and Weight Management: A Review of Current Evidence and Future Directions

**DOI:** 10.1002/fsn3.71801

**Published:** 2026-04-23

**Authors:** Ayesha Rehman, Noman Ali, Muhammad Rizwan Tariq, Shinawar Waseem Ali, Azeem Mushtaq, Waseem Safdar, Abdikhaliq Mursal Yusuf

**Affiliations:** ^1^ Department of Public Health University of the Punjab Lahore Pakistan; ^2^ NIFSAT University of Agriculture Faisalabad Faisalabad Pakistan; ^3^ Department of Food Sciences University of the Punjab Lahore Pakistan; ^4^ Department of Biosciences National University of Medical Sciences Islamabad Pakistan; ^5^ Kaalo Aid and Development Organization, KAALO Aid HQ Garowe, Puntland Somalia

**Keywords:** dysbiosis, gut microbiome, obesity, SCFAs

## Abstract

Obesity has become an emerging challenge all over the world. In 2022, one in eight people was living with obesity. It is most common in adults and children. It is due to an imbalance between energy consumption and utilization. However, the human gut microbiome regulates energy metabolism, a complex ecosystem of microorganisms residing in the gastrointestinal tract, which stimulates hormone production, produces various metabolites, and interacts with brain responses involved in maintaining energy balance in the body. Dysbiosis, characterized by an imbalance in microbial composition, has been linked to increased energy harvesting, impaired bile acid metabolism, chronic low‐grade inflammation, impaired appetite regulation, and excessive intake, ultimately leading to obesity. The primary focus of this review is to discuss the current understanding of the roles of diet, exercise, pharmacological agents, and surgery in shaping gut microbial communities and host physiology. Dietary interventions, such as the use of probiotics, prebiotics, high‐fiber diets, ketogenic diets, and intermittent fasting, modulate microbial metabolites like short‐chain fatty acids, which play a crucial role in regulating energy balance and inflammation, thereby contributing to the prevention of weight gain. Physical activity induces positive changes in gut microbiota, enhancing metabolic adaptability and supporting immune system regulation. Pharmacological treatments, especially anti‐obesity and anti‐diabetic drugs, have both direct and microbiota‐mediated effects on weight and glucose metabolism. Similarly, surgery leads to significant changes in gut microbiota, which play a role in long‐term enhancements in metabolic health. So, this review aims to discuss various weight management approaches targeting the gut microbiome, drawing on current studies. However, these interventions require further investigation for their effectiveness.

## Graphical abstract

The gut microbiome is critical in controlling host metabolism and body weight. Gut microbial composition and functionality are regulated by external factors such as diet, exercise, medications, and surgical procedures. The microbiome, in turn, affects energy balance, fat storage, and metabolic processes, leading to the development of obesity and weight control. These interactions are bidirectional and dynamic, highlighting the microbiome as a therapeutic target.

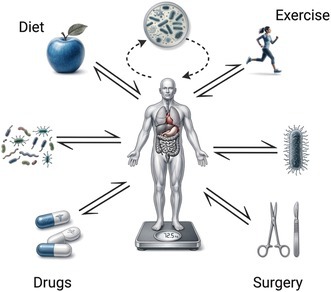

## 1 Introduction

**FIGURE 1:**
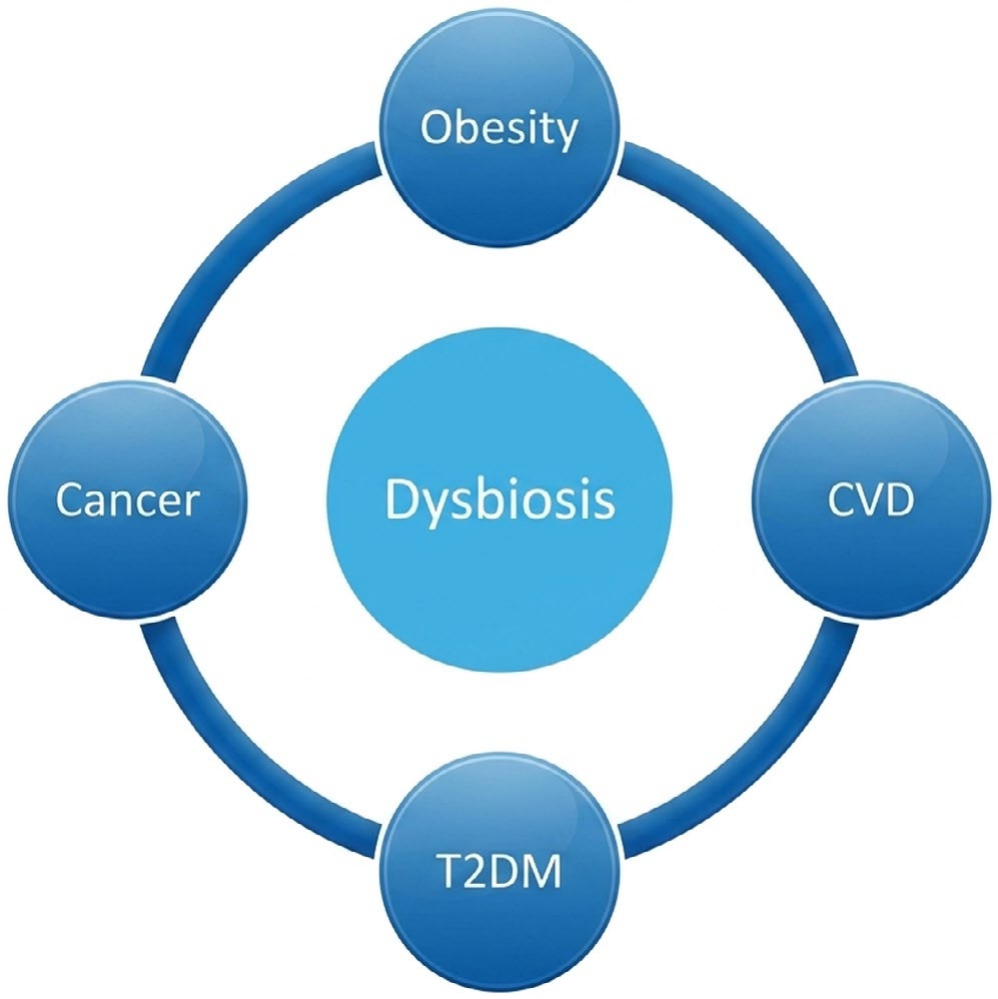
Dysbiosis‐associated diseases.

Obesity is the most common disease characterized by excessive accumulation of adipose tissue, which can have adverse effects on health (Timothy Garvey 2018). Adipose tissue serves as a crucial storage site for surplus nutrients (Parra‐Peralbo et al. 2021). A recent report published in The Lancet indicates that, as of 2022, over 1 billion individuals globally are living with obesity (WHO 2024). Individuals with a body mass index (BMI) greater than 40 kg/m^2^, exhibiting excess adiposity that is readily apparent, require no further confirmation (Caballero 2019). Obesity is influenced by various factors, including environmental, lifestyle, and genetic factors, indicating that it is a multifaceted condition (Verde et al. 2024). A spectrum of comorbidities, including type 2 diabetes (T2D), cardiovascular disease, and certain cancers, is associated with obesity, which significantly reduces life expectancy and quality of life, as depicted in Figure 1 (Zhang et al. 2023). Obesity prevalence is rising in both low and high‐income countries. Evidence suggests that increased accessibility to junk food outlets and local stores offering packaged foods with excessive sugar and fat content contributes to this trend (Creatore et al. 2016).

A GI microbiome is a complex community of thousands of billions of microorganisms residing within the gastrointestinal tract (Roychoudhuri et al. 2025). This microbiome represents a relatively stable microbial ecosystem within an individual (Johnson et al. 2019). It plays a crucial role in human health by supporting metabolic processes, physiological functions, and nutrient absorption (Duman and Karav 2025). These important microbes perform critical structural, protective, and metabolic functions. The protective function involves the formation of the humoral component within the gut's mucosal immune system (Zhou et al. 2022). The structural role includes strengthening the intestinal barrier and enhancing apical tight junction integrity (Barbara et al. 2021). The metabolic activity of the GI‐microbiome is both adaptable and renewable (Bocci 1992). Dysbiosis, defined as the disruption of the gut microbial community, is associated with IBD (Santana et al. 2022), IBS (Wang et al. 2020), obesity, and a host of other conditions (Amabebe et al. 2020).

Mechanistically, the gut microbiome influences energy homeostasis through a variety of pathways, including modulation of dietary energy extraction (Corbin et al. 2023), SCFA production (Fusco et al. 2023), integrity of the gut barrier (Kocot et al. 2022), bile acid metabolism (Collins et al. 2023), and appetite regulation via gut‐brain signaling (Han et al. 2021). Interventions targeting the microbiome have emerged as significant treatment methods. Prebiotics, probiotics, fecal microbiota transplantation (FMT), dietary modifications, and next‐generation microbial therapies are potential modalities for the restoration of microbial balance and the management of weight (Santos‐Paulo et al. 2022). However, further study into the potential impact of these interventions on weight management is still needed. In this review, we will discuss the current scientific evidence linking the gut microbiome to obesity and weight management.

Dysbiosis is associated with obesity, which further contributes to its metabolic complications, such as CVD, T2DM, and Cancer.

## 2 Characterization of Gut Microbiome in Lean vs. Obese Individuals

The combination of bacteria, fungi, viruses, and eukaryotes, along with the tissues in which they reside, is collectively referred to as the microbiota. The specific community of microbiota within an individual is known as the microbiome (Leonard and Toro 2023). This microbial consortium, comprising over 10^3^ microorganisms, is primarily dominated by the phyla Firmicutes and Bacteroidetes (Dezfouli et al. 2025). Innovations in recent DNA sequencing technologies have made it feasible to detect and characterize these microbial communities, including their alternation within and between individuals and groups. The complex compound, such as *Lactarius hatsudake* Tanaka, which is a popular edible mushroom known for its pleasant flavor and health benefits, is digested by the gut microbiome. Research indicates that the direct digestion of LHP by the human digestive system is not possible; however, the gut microbiota can predominantly break it down into fatty acids, leading to a decrease in molecular weight, pH, and carbohydrate content. In addition to this, LHP explores the increase of beneficial bacteria (*Bifidobacterium*, *Faecalibacterium*, *Lactobacillus*) while inhibiting the growth of harmful *Escherichia* and *Shigella* (Yang et al. 2025).

The gut microbiota affects immune cell maturation and the synthesis of immune‐modulating molecules (Missal et al. 2025). These are also involved in the metabolism of bile acids and amino acids, as well as in vitamin synthesis. The human microbiome exhibits significant inter‐individual and inter‐site diversity, shaped by genetics, age, diet, lifestyle, and environmental exposures (Al‐Matouq et al. 2025). An altered gut microbiota, in conjunction with lifestyle factors, may contribute to the development of childhood obesity (Bervoets et al. 2013). While the connection is complex and not fully understood, research suggests several potential mechanisms. Obese individuals experience a reduction in gut microbial diversity, leading to disruptions in healthy microbial functions (Chanda and De 2024). Studies have shown a lower ratio of Firmicutes to Bacteroidetes in obese individuals compared to normal‐weight individuals, contradicting earlier findings that suggested a higher ratio in obesity (Zárate‐Córdova et al. 2025).

**FIGURE 2:**
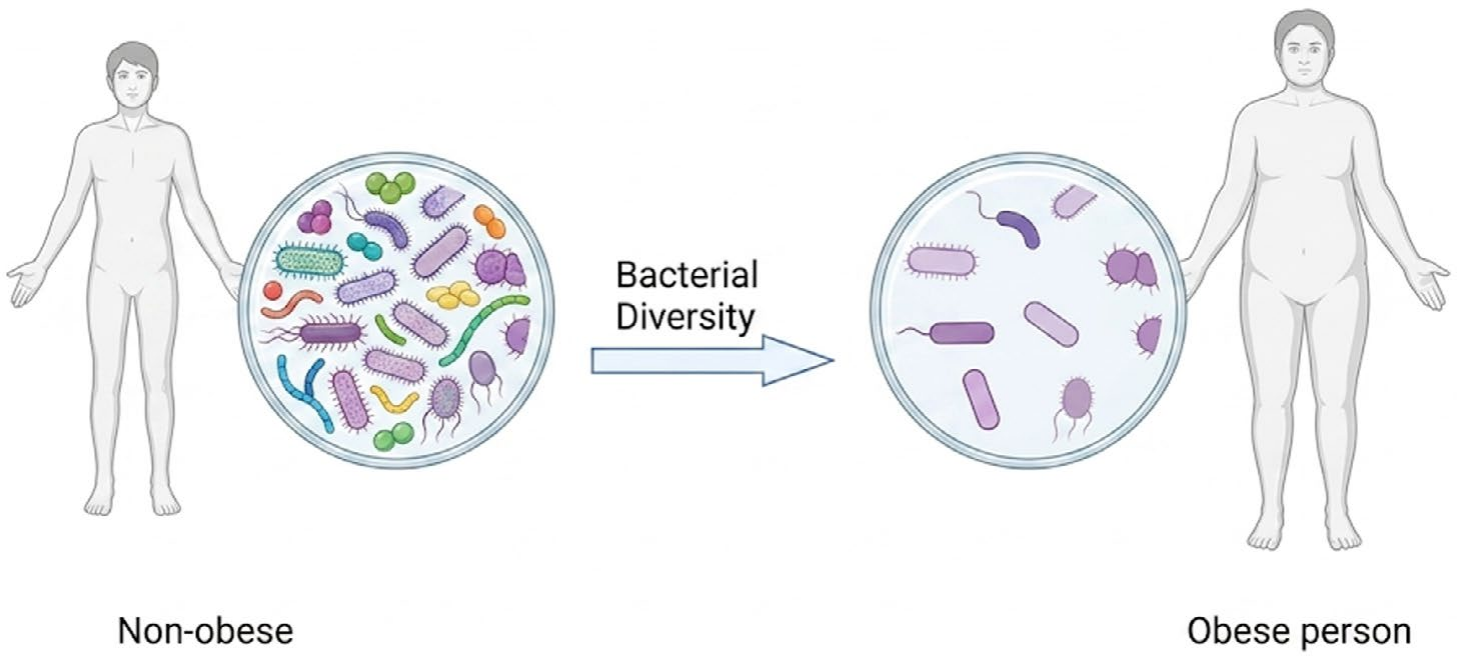
Gut microbial diversity difference between obese and non‐obese individuals.

Some studies have previously proposed that an increase in the Firmicutes‐to‐Bacteroidetes ratio in obese individuals and animals facilitates greater energy extraction from food, associated with greater calorie absorption and weight gain (Magne et al. 2020). However, more recent research presents a nuanced view. Some bacterial species, such as *Akkermansia muciniphila* , are more prevalent in lean individuals and play a crucial role in maintaining intestinal health and modulating host metabolism, thereby preventing inflammation (Zhang et al. 2025). Obese individuals often exhibit a decrease in SCFA‐producing bacteria (e.g., Alistipes species, *Odoribacter splanchnicus* ) and a depletion of bacteria that promote gut barrier integrity ( *Akkermansia muciniphila* , *Bifidobacterium longum* ) as demonstrated in Figure 2 (Chanda and De 2024). Infants are born with a sterile gut, which is rapidly colonized by bacteria from the mother and the environment. The gut microbiota composition remains unstable until approximately 3–4 years of age, when it reaches maturity. Gut colonization offers two primary benefits: it educates the immune system and enhances tolerance to microbial immune determinants (Den Besten et al. 2013).

## 3 Obesity‐Related Alterations in Gut Microbiota

### 3.1 Energy Harvesting and SCFA Production

**FIGURE 3:**
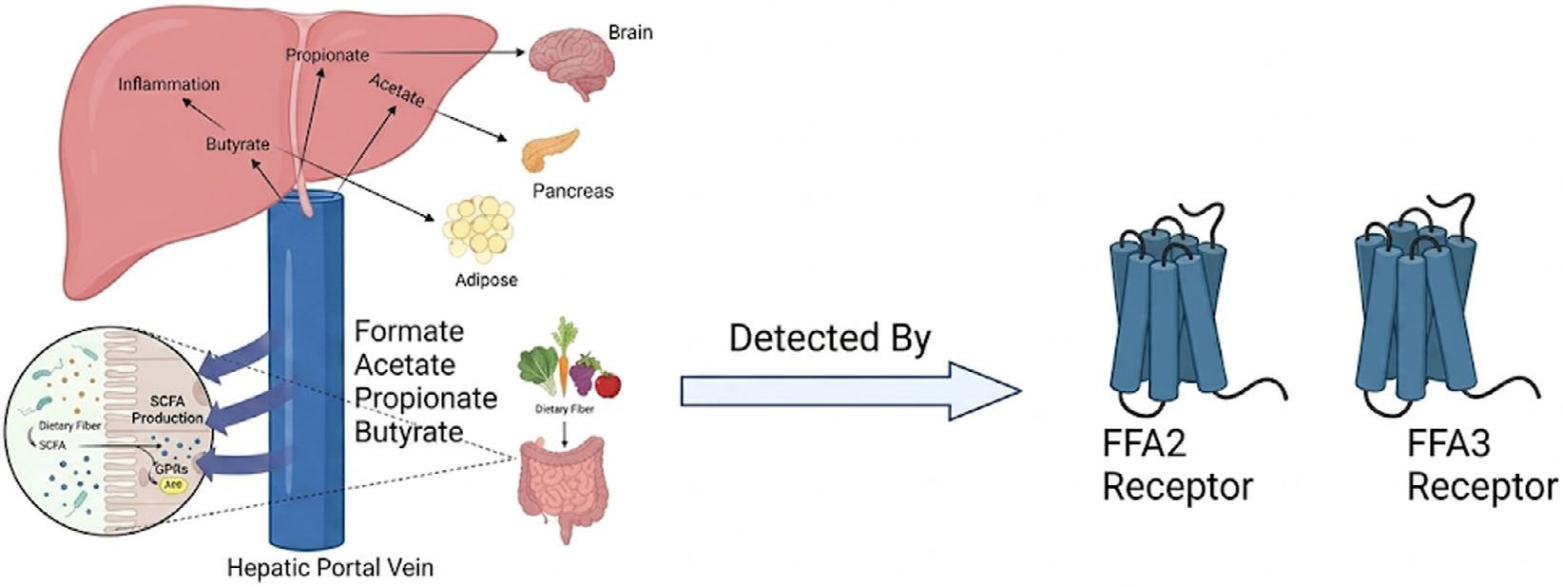
SCFAs production and its targeting function.

It has been demonstrated that the composition of gut microbial flora in individuals with obesity differs from that of lean individuals, characterized by a higher relative abundance of Firmicutes and a lower relative abundance of Bacteroidetes. Such changes in microbial populations increase energy harvest by the obese‐type microbiota. This finding was further verified experimentally in GF mice, where greater fat deposition was observed when gut microbiota from an obese donor was transplanted into mice (Turnbaugh et al. 2006), compared to that from lean donors, confirming the critical role of gut microbiota in obesity (Meijnikman et al. 2020). Nonetheless, the relationship between microbiota structure and energy harvesting is complex and not simply a matter of balancing the various dominant phyla (Murphy et al. 2010). Short‐chain fatty acids (SCFAs) are detected by specific membrane‐bound receptors, FFA2 and FFA3, which are distinctly distributed throughout the gastrointestinal tract, as (Figure 1) presented in Figure 3. These receptors play key roles in regulating gut motility, hormone release, maintenance of the epithelial barrier, and immune cell function (Priyadarshini et al. 2018). The products of fermentation of non‐digestible carbohydrates, such as short‐chain fatty acids (SCFAs), significantly influence the gut‐brain axis and thus the host's energy homeostasis (Barrea et al. 2019). These primary metabolites, including formate, acetate, propionate, and butyrate, are synthesized by a wide range of bacterial groups as presented in Table 1 (Morrison and Preston 2016). Animal and human studies have demonstrated that acetate positively influences energy and nutrient metabolism by stimulating the release of gut hormones, including glucagon‐like peptide‐1 (GLP‐1) and peptide YY (PYY). These hormones help regulate appetite, reduce overall fat breakdown (lipolysis), lower levels of inflammatory cytokines, and enhance both energy use and fat burning (González Hernández et al. 2019). Propionates have anti‐inflammatory properties (Mandaliya et al. 2021). Butyrate exhibits a stronger correlation with the presence of particular specific bacterial species, while propionate is produced or influenced under a broader range of conditions. The increase in these bacteria could thus enable the greater production of reportedly beneficial metabolites, facilitating targeted health interventions (Kircher et al. 2022). Propionate will act on the sympathetic nervous system (SNS) via its GPR41 receptor, driving energy expenditure and countering weight gain (Kimura et al. 2011). Whereas increased acetate levels would stimulate the parasympathetic nervous system to release insulin, promote ghrelin production, and lead to overeating as a contributor to weight gain and obesity‐related issues. Thus, regulation of acetate production may offer new therapeutic strategies for obesity treatment (Perry et al. 2016). Butyrate has been shown to inhibit energy intake while promoting fat oxidation in brown adipose tissue, facilitating weight maintenance and metabolic improvement, even when individuals are challenged with a high‐fat diet (Lai et al. 2018). While fecal SCFA levels are generally reported to be higher in the obese than the non‐obese population, in contrast, no significant intergroup differences in the gut bacterial composition could be resolved, leading to further investigations to clarify whether high SCFA levels work as a causative influence in obesity or as a mere result due to other factors (Kim et al. 2019) (Figure 2).

**TABLE 1:** Microbial metabolites involved in obesity and weight regulation.

Metabolites	Producing bacteria	Effect on host metabolism	Evidence (animals/humans)
Acetate Hosmer et al. (2023)	*Bacteroidetes* spp. *Prevotella* spp. *Bifidobacterium* Akkermansia	*Appetite control* (stimulates GLP‐1, PYY secretion), Enhance lipogenesis (substrate for fatty acid & cholesterol synthesis), *Regulate inflammation* (reduces systemic pro‐inflammatory cytokines)	Human and animal studies (higher acetate in obese individuals)
Propionate Wang et al. (2024)	Bacteroidetes, Firmicutes, *Veillonella parvula*	Mucosal immunity modulation, appetite regulation, anti‐inflammatory, anti‐lipogenic, and cholesterol‐lowering	Human intervention studies Study of Animal models; human observational studies
Butyrate Zhang et al. (2021)	Firmicutes, Bacteroidetes, *Roseburia spp*	Thermogenesis, lipid and glucose metabolism, appetite, and inflammation	Most of animal studies and limited human data

SCFAs produced inside the intestine enter the liver via the hepatic portal vein circulation and contribute to maintaining liver health by acting on macrophages and hepatocytes that reside in the liver. Each SCFA interacts with specific organs, such as the brain, pancreas, and adipose tissue, to support their function. SCFAs are detected by receptors like FFA2 and FFA3, which affect intestinal function.

### 3.2 Gut Hormone and Appetite Regulation

The gut microbiome influences obesity by regulating gut hormones that control appetite and energy metabolism (Wren and Bloom 2007). The short‐chain fatty acids (SCFAs) trigger the release of incretin hormones, peptide YY (PYY), and glucagon‐like peptide‐1 (GLP‐1) through G‐protein coupled receptors (GPCRs) on the hollow enteric endocrine L‐cells in the intestine. These SCFAs are produced by the dominant gut microbiome phyla Bacteroidetes and Firmicutes (Tolhurst et al. 2012). GLP‐1 maintains blood sugar homeostasis by enhancing insulin release and inhibiting glucagon secretion upon nutrient ingestion (Andersen et al. 2018), delays gastric emptying, and suppresses appetite (Klausen et al. 2022). GLP‐1 receptor agonists (GLP‐1RAs) promote satiety by stimulating GLP‐1R neurons located in the dorsomedial hypothalamus (DMH) within the brain that are linked to hunger‐regulating neuronal circuits. The regulation of food intake via this mechanism may provide possible therapeutic targets for obesity treatment (Kim et al. 2024).

In contrast, obese individuals exhibit reduced meal‐induced secretion of GLP‐1 compared with lean controls; the clinical relevance of this observation about the development of obesity requires further investigation (Krieger 2020). After food consumption, the gastrointestinal tract releases PYY. Its level starts to rise, and within 1–2 h it attains the optimum level. After that, it continues to be elevated for a longer duration, which indicates that its specific role is in giving a feeling of fullness (as a satiety factor) rather than as a food intake terminator, and protects from weight gain (Chandarana et al. 2009). Dysbiosis, which is a modulation in the gut microbiome, can affect the production of SCFAs and the release of gut microbiome, which ultimately leads to excessive food intake and weight gain (Batterham et al. 2002).

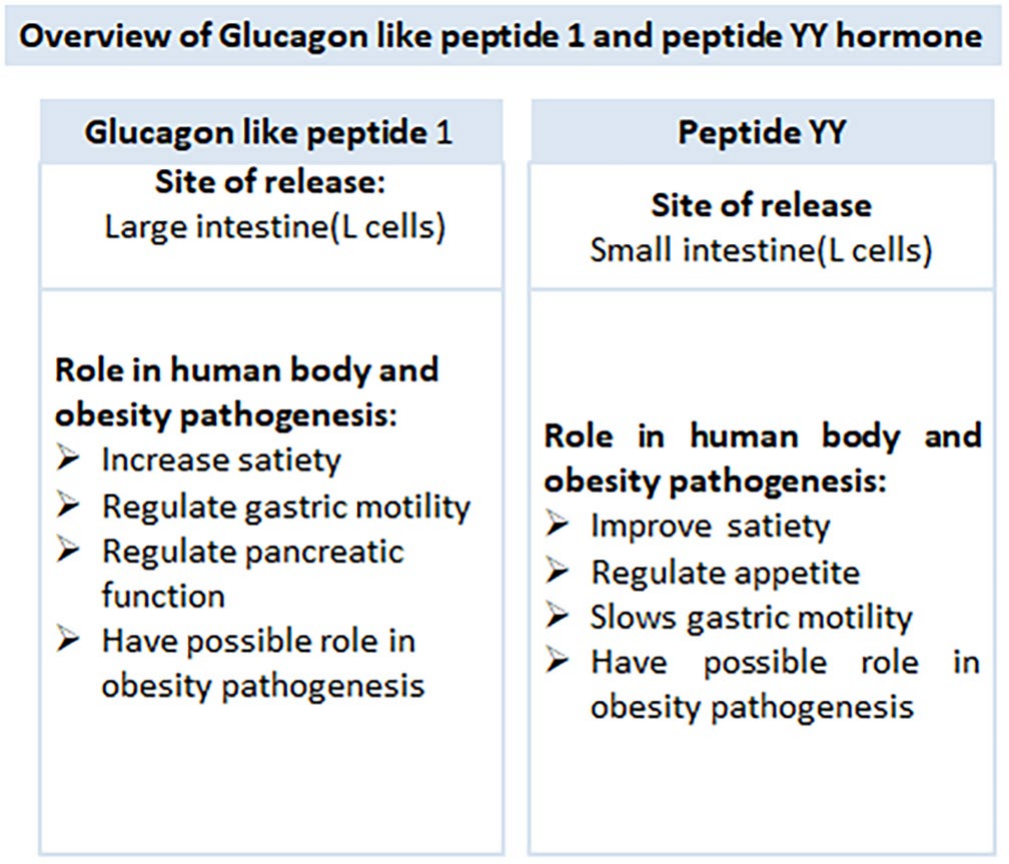

### 3.3 Gut‐Brain Axis

**FIGURE 4:**
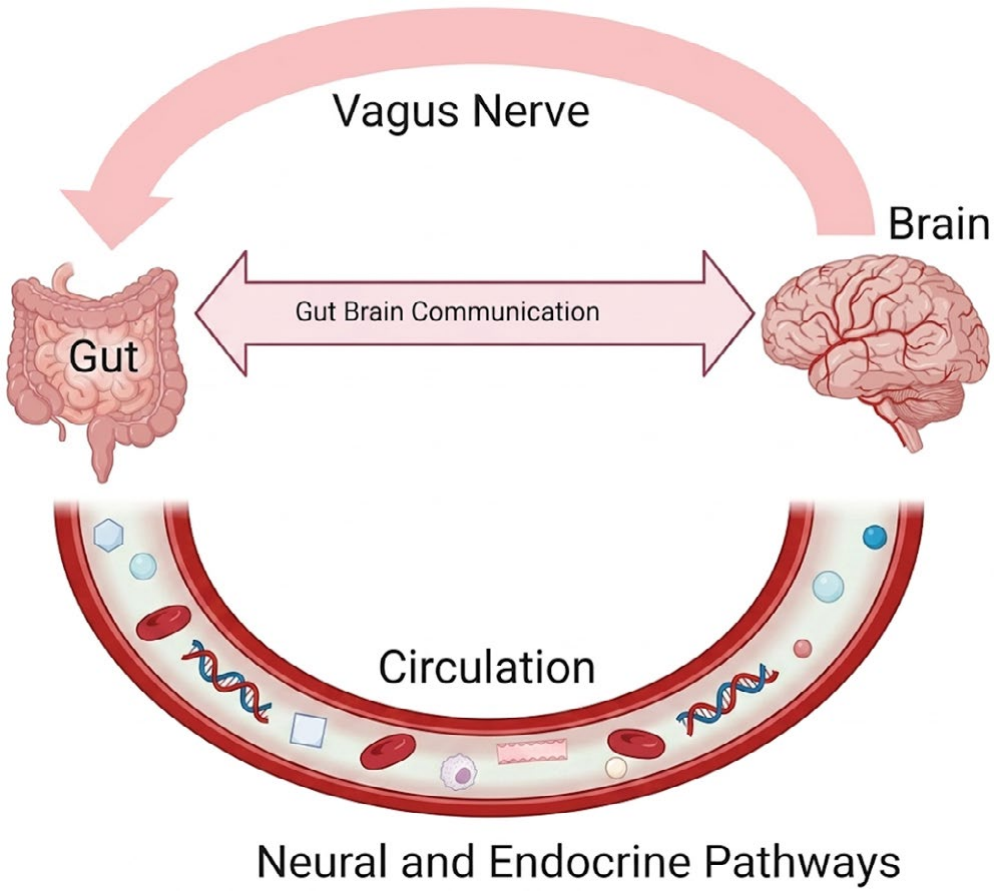
Gut‐brain axis.

The gut microbiota plays a significant role in complex and dynamic metabolic processes that influence host brain function, immune responses, gender‐specific metabolism, and the symbiotic relationship it has with the host. These can also stimulate the synthesis of vital metabolites, neurotransmitters, and other neuroactive substances that influence the progression and treatment of central nervous system diseases. Gut‐brain signaling is a pathway and the mechanism of communication between the gut and the brain, as illustrated in Figure 4 (Lu et al. 2024). The gut interacts with the central nervous system to convey information about nutritional status through various mechanisms, including the enteric nervous system (ENS), the vagus nerve (VN), and enteroendocrine cells (EECs). Microbial‐produced compounds can modulate these signaling pathways (van Son et al. 2021). The signals transmitted by vagal pathways can produce either anxiogenic or anxiolytic effects, depending on the type of stimulus (Forsythe et al. 2014).

The GBA is a complex communication pathway that encompasses the enteric nervous system, the vagus nerve, neurotransmitters, gut hormones, and gut microbiota, which together regulate digestion, metabolism, mood, and behavior. Current research highlights the GBA's role in neurotransmitter synthesis, gut microbiota composition, immune modulation, stress response, and vagal nerve signaling, underscoring its significance for mental health. Furthermore, GBA dysregulation can lead to altered appetite, metabolic dysfunction, and alterations in gut microbiota composition, contributing to the development and progression of obesity (Zheng et al. 2023). One mechanism by which this occurs involves the production of SCFAs, primarily acetate (C2), propionate (C3), and butyrate (C4), which are the most prevalent anions in the colon and are generated through the fermentation of dietary fiber and resistant starch by gut bacteria (Portincasa et al. 2022). These metabolic products stimulate the colonic L cells to secrete peptide YY (PYY) and glucagon‐like peptide‐1 (GLP‐1) hormones, known to suppress appetite and induce satiety in both animal models and humans (Stanley et al. 2004). The imbalance in the gut microbiome known as dysbiosis can disrupt SCFA production, which is crucial for regulating hormone secretion. This disruption can ultimately affect a key path between the gut and brain known as the vagus nerve, particularly the hypothalamus, which is involved in regulating hunger and energy expenditure (Longo et al. 2023).

This diagram illustrates the communication link between the brain and the gut, which is influenced by various pathways, including endocrine, enteric nervous system, and vagus nerve pathways.

### 3.4 Gut Barrier Integrity and Inflammation

Over the past two decades, substantial evidence has accumulated to establish a link between obesity and inflammation. Adipose tissue functions as an energy reservoir, but it is also an active metabolic and endocrine organ that secretes adipokines, chemokines, and pro‐inflammatory cytokines, including tumor necrosis factor alpha (TNF‐α), interleukin‐6 (IL‐6), plasminogen activator inhibitor 1 (PAI‐1), and leptin, thereby promoting a state of chronic inflammation. This inflammation can be exacerbated by various external factors, such as diet or the use/abuse of antibiotics, which can alter the gut microbiome (Petraroli et al. 2021). The mucosal surface layer serves as an essential barrier across the epithelial membrane, separating luminal contents from the outside environment. These epithelial cells not only perform protective functions but also facilitate nutrient absorption, requiring specific barrier permeability. The mucosal epithelium also plays a central role in coordinating immune responses to foreign substances, including dietary antigens and microbial metabolites.

Recent advancements have shown that external stimuli can activate multiple mechanisms regulated by the intestinal mucosal barrier system. A key component of this epithelial boundary is the physical intracellular structure called tight junctions. TJ consists of various transmembrane proteins that are connected to cytoplasmic adaptors, aiding in the attachment of adjacent cells. Disturbance of this barrier can directly affect both healthy and disease states, as dysfunction in the barrier has been linked to the onset of inflammation and pathological effects associated with metabolic disorders (Panwar et al. 2021). Intestinal barrier dysfunction, along with bacterial translocation and its byproducts, has been identified as a key factor in the pathophysiology of obesity. Impaired barrier integrity can increase intestinal permeability, allowing microbiota‐derived endotoxin, such as lipopolysaccharides, to enter systemic circulation. The presence of these immune‐stimulatory ligands is recognized by Toll‐like receptor 4 (TLR4), triggering pro‐inflammatory reactions that promote insulin resistance. Therefore, targeting intestinal barrier function represents a promising therapeutic strategy for preventing and treating obesity (Ma et al. 2020).

Effective maintenance of the barrier requires coordinated interactions between epithelial cells and the microbiota, supporting cross‐talk between the microbiome and the intestinal epithelium, thereby establishing a complex interdependence essential for maintaining intestinal homeostasis. The gastrointestinal tract, which is continually exposed to various stimuli, plays a crucial role in maintaining the host's overall homeostasis. Billions of microorganisms populate the gut, collectively known as the gut microbiota, and engage in a mutualistic relationship with the host. While the microbiota is generally considered beneficial, it, along with pathogenic microorganisms, represents a continuous threat to the host. Different populations of epithelial cells form the primary chemical and physical barrier against external factors, acting as an interface between luminal microbes and the immunocompetent cells in the lamina propria (Gieryńska et al. 2022).

### 3.5 Bile Acid Metabolism

**FIGURE 5:**
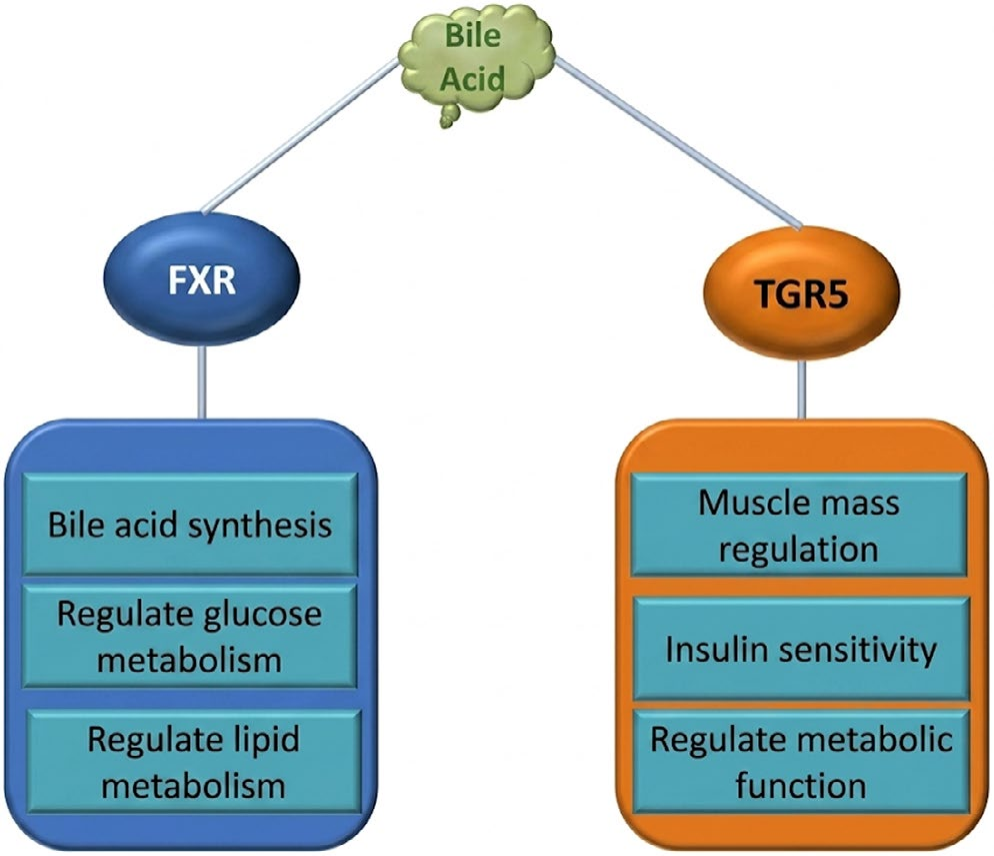
Functions regulated by bile acid.

Bile acids, derived from cholesterol metabolism, are crucial for liver lipid regulation. Synthesized in the liver and modified by gut microbiota into secondary bile acids, these compounds undergo enterohepatic circulation, a process tightly regulated by negative feedback to maintain homeostasis and prevent hepatotoxicity (Peng et al. 2025). The complex interaction between bile acids and the gut microbiota significantly influences host metabolism. Bile acids function as signaling molecules, activating receptors such as the farnesoid X receptor (FXR) and TGR5, which regulate diverse metabolic pathways, including bile acid synthesis, glucose and lipid metabolism, and even muscle mass regulation, as (Table 1) outlined in Figure 5. Furthermore, bile acids influence gut microbial composition, highlighting the mutual relationship between these two factors. Disruptions in this delicate balance can alter bile acid profiles and contribute to various disease states (Ramírez‐Pérez et al. 2018) (Figure 4).

The farnesoid X receptor (FXR), a nuclear receptor activated by bile acids, plays a pivotal role in bile acid homeostasis. FXR activation in the liver suppresses bile acid synthesis by inducing SHP, which in turn inhibits CYP7A1, the rate‐limiting enzyme in this process. In the intestine, FXR triggers the production of FGF19 (FGF15 in rodents), which signals back to the liver to further inhibit bile acid biosynthesis. Beyond bile acid regulation, FXR also modulates glucose and lipid metabolism and has been implicated in inflammation and fibrosis, making it a potential therapeutic target for metabolic disorders such as NASH and type 2 diabetes. Additionally, bile acids activate the TGR5 receptor, influencing muscle mass and other metabolic functions (Sato 2025). The gut microbiota's ability to transform bile acids into secondary forms further complicates this signaling pathway, impacting both receptor activation and microbial community structure, ultimately influencing host physiology (Di Ciaula et al. 2022).

Bile acid synthesis occurs in the liver. It activates FXR and TGR5 receptors, which perform important functions in the body, such as glucose and lipid metabolism, muscle mass regulation, and regulating other metabolic functions.

## 4 Microbiome‐Targeted Interventions for Weight Management

### 4.1 Dietary Interventions

#### 4.1.1 Probiotics

**FIGURE 6:**
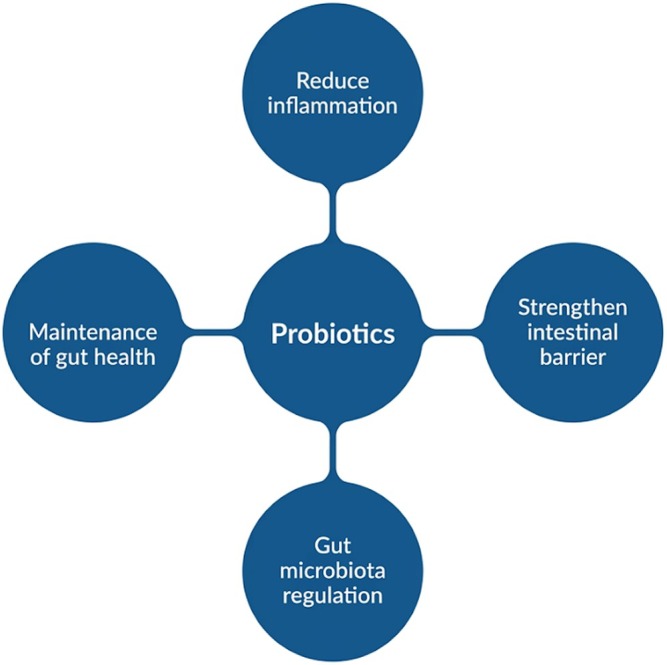
Probiotics benefits.

The disruption of normal physiological processes can be linked to an imbalance in the gut microbiota, often associated with obesity, which impairs the population of beneficial microorganisms (Ben Othman et al. 2023). These microbial alterations further contribute to disruption in energy homeostasis, lipid metabolism, hormonal regulation, and the intensification of low‐grade systemic inflammation, as represented in Figure 6. Probiotic supplementation has demonstrated promise in modulating the gut microbiota, potentially reducing intestinal permeability, inflammation, and metabolic dysregulation, thereby fostering conditions that may support weight reduction (Marques et al. 2020).

Probiotics are sustainable microorganisms, predominantly from the genera *Lactobacillus* and *Bifidobacterium*, that are essential for preserving or reestablishing the equilibrium of the gut microbiota, thereby controlling conditions associated with alteration in microbial composition. These probiotics help to strengthen the intestinal epithelial barrier, prevent the establishment of pathogenic microbes, and support the maintenance of gastrointestinal health (Takahashi, n.d.). Several mechanisms of action for probiotics have been identified; however, the primary mechanism by which they can affect the host is their regulatory effect on the gut microbiota.

Understanding the benefits of microbiota stability suggests that it is sensible to manage gut microbiota composition through probiotic consumption (Nourizadeh et al. 2022). As time passed, the use of probiotics as a food supplement has rapidly expanded due to their beneficial effects on gut homeostasis (Sakandar and Zhang 2021). In a randomized controlled trial, patients with obesity were treated with probiotic supplementation, and a notable decrease in body fat, BMI, and waist circumference was observed, with greater reductions when treatment was administered for a longer duration (Cao et al. 2020). Another Multicenter Randomized Placebo‐Controlled Study showed a significant reduction in blood sugar levels and appetite in overweight patients who were given a specific strain, *Hafnia alvei* HA4597, in combination with a mildly low‐calorie diet and routine exercise for 12 weeks (Déchelotte et al. 2021). It is recommended that homogeneous populations, in terms of sex and age, should also be considered when conducting studies.

In addition to this, future trials carried out in the absence of weight loss interventions (such as dietary recommendations for weight loss and physical activity programs) would be more favorable for analyzing the specific effect of the strains (Álvarez‐Arraño and Martín‐Peláez 2021). The use of prebiotics and probiotics, combined with lifestyle interventions, appears promising for managing obesity, particularly when linked to the enhancement of metabolic parameters and improvement of associated psychiatric conditions (Ben Othman et al. 2023). Further studies are required before probiotics can be recommended as a therapeutic approach for these patients (Perna et al. 2021). The effectiveness of probiotics is influenced by several factors, including the specific strain used, the dosage administered, and the delivery method. Furthermore, external conditions, stability during storage, and potential interaction with other additives can significantly affect their therapeutic outcomes (Naeem and Bourassa 2025). Meta‐analyses of randomized controlled trials (RCTs) have shown modest but significant reductions in body weight and BMI with probiotic supplementation, though effects are strain‐ and dose‐specific (e.g., *Lactobacillus gasseri* , *Bifidobacterium breve* ) (Cao et al. 2020).

#### 4.1.2 Prebiotic

The gut microbiota plays a crucial role in regulating metabolic processes, immunological defense, and inflammation within the host. However, changes in gut microbiota composition have been linked to the development of obesity and obesity‐related disorders, as this altered gut microbiome has been associated with increased energy extraction from non‐digestible dietary carbohydrates, increased gut permeability, and insulin resistance. Based on existing data, gut microbiota regulation can be achieved through either dietary intervention or the administration of prebiotics (Sankararaman et al. 2023). Prebiotics are non‐digestible dietary components that selectively promote the growth and/or activity of beneficial probiotic microorganisms in the GIT, thereby promoting human health. Typically, these prebiotics are dietary fibers that are not digested in the small intestine. Upon reaching the large intestine, they undergo fermentation by the intestinal microbiota, producing SCFAs. Examples of prebiotics include inulin, oligosaccharides, disaccharides, monosaccharides, and short‐chain fatty acids (Erkmen 2018). Prebiotics exert beneficial effects on the regulation of metabolic diseases, such as obesity and type 2 diabetes mellitus, by modulating the gut microbiota. Many innovative food processing techniques, such as enzyme‐modified prebiotics and probiotic‐fermented natural foods, have been developed to enhance these beneficial effects. Moreover, in vitro and in vivo studies have shown that one of the most direct mechanisms of prebiotics is to alter the gut microbiome composition, which helps decrease metabolic disease by regulating the abundance of Lachnospiraceae, Akkermansia, Proteobacteria, and Rikenellaceae (Li et al. 2021). Prebiotic consumption has been shown to improve the composition of the gut microbiota, leading to enhanced activity of enteroendocrine cells that secrete hormones regulating food intake, energy balance, and body weight. Prebiotics, commonly found in fruits and vegetables, offer numerous health benefits. These include lowering low‐density lipoprotein levels and aiding the absorption of essential minerals such as calcium, magnesium, zinc, and iron due to their ability to bind with these minerals. This binding mechanism may reduce mineral absorption in a specific region of the small intestine.

However, further research is needed to fully understand the mechanism and impacts of prebiotics on human health (Dharmatti 2025). In a longitudinal intervention study, patients undergoing weight loss were given prebiotics in conjunction with a low‐carbohydrate, adequate‐fiber, and adequate‐protein diet, with a focus on reducing energy intake. After 3 months, a statistically significant difference was observed in anthropometric and body composition parameters between pre‐ and post‐follow‐up measurements. At the end of the study, all these parameters decreased due to a significant shift in gut microbiome composition, characterized by increased relative abundances of *Lactobacillus*, Bifidobacteria, and Bacteroidetes, and decreased relative abundances of Firmicutes, as well as a decrease in the Firmicutes/Bacteroidetes Ratio (Hassan et al. 2024). While in vivo and in vitro studies in animals and humans have shown that the growth of *Bifidobacterium* and *Lactobacillus* species is enhanced by prebiotics, these prebiotics trigger a wide‐ranging shift in the Bacteroidetes and Firmicutes phyla. The mechanism by which carbohydrates interact with GM and the host is still not fully understood, and further research is needed to unravel it (Megur et al. 2022). Prebiotics (e.g., inulin, oligosaccharides) consistently enhance gut microbial diversity and SCFA production, with beneficial effects on satiety hormones and weight regulation; however, long‐term adherence and sustainability remain uncertain (Hassan et al. 2024).

#### 4.1.3 Symbiotic

Alteration in the gut microbiota composition brought about by synbiotics, which consist of a combination of probiotics and prebiotics, might lead to small reductions in body weight and waist circumference (Hadi et al. 2020). They have the potential to create considerable synergistic improvements in the probiotic and prebiotic beneficial effects (Cerezuela et al. 2011). Synbiotic treatments are more effective in altering gut microbiota compared to prebiotics or probiotics alone (Saulnier et al. 2008). Research has shown that synbiotic intake is associated with a significant shift in fecal flora, characterized by an increase in the number of Bifidobacteria and Lactobacilli, accompanied by a decrease in *Clostridium Perfringens* (Rafter et al. 2007). Targeting the gut microbiota with synbiotics is emerging as a promising strategy within a comprehensive nutritional approach to reduce obesity rates. Weight loss after low‐carbohydrate, high‐protein diets can be significant, but it has also been associated with potentially adverse health effects due to the excessive fermentation of undigested protein inside the colon and consequent alterations in the composition of the gut microbiome (Sergeev et al. 2020). Systematic reviews suggest that symbiotics have synergistic effects on weight and waist circumference, but most studies are limited by small sample sizes and short durations (Hadi et al. 2020).

#### 4.1.4 Intermittent Fasting

Intermittent fasting provides a strategy for improving weight and metabolic profiles. There are several methods: alternate‐day fasting, in which 24‐h fasts are done every other day, and the 5:2 approach, which involves fasting for two 24‐h periods over the week alongside a very low‐calorie diet on two other non‐consecutive days. Those fasting periods determine whether it becomes continuous. Another popular method is time‐restricted feeding, which involves daily fasting, usually with a 6‐h feeding window. This often means breakfast and dinner before 3 p.m., which results in 14–18 h of fasting (Anton et al. 2018). Intermittent fasting protocols enforce an alternating fed, post‐absorptive, and fasting metabolic state. Insulin is active during the fed state, with glucose serving as the primary energy source. Glucagon, on the other hand, is activated during fasting, signaling the body to utilize hepatic glycogen as an energy source.

The metabolic transition, which corresponds to negative energy balance, occurs only after glycogen stores are replenished, and fatty acid metabolism begins, usually within 12 h or more postprandially. This metabolic transition from glucose to ketone utilization may be considered an evolutionary adaptation as it shifted metabolism from lipid/cholesterol synthesis and storage to the mobilization of fats via fatty acid oxidation and ketogenesis, thereby protecting muscle mass and function. Accordingly, it is proposed that intermittent fasting, which causes this metabolic switch, may improve body composition in overweight individuals; however, this has yet to be investigated (Vasim et al. 2022). Motivated by positive metabolic changes driven by periodic conversion of fat into ketones, these intermittent feeding schedules could lead to significant weight reduction on a large scale, as they almost always result in a negative caloric balance. Around that point, lipolysis of triglycerides stored in adipose tissue converts them into fatty acids and glycerol, which fuel extended fasting periods. After which, these fatty acids are converted into ketone bodies in the liver. Considering that ketone bodies become the primary energy substrate for many tissues and the brain during fasting, ketogenesis becomes functional only when subjects are fasting or near fasting (Anton et al. 2018).

#### 4.1.5 Caloric Restriction

Caloric restriction (CR) encompasses dietary interventions that involve reduced energy intake while maintaining adequate nutrition and has been shown to prolong both health span and lifespan in rodent and primate models (Most et al. 2017). Multiple experimental and clinical studies in humans and rodents have documented a range of beneficial effects of CR, including weight and fat loss, protection against hyperglycemia and hyperinsulinemia, and reduced inflammation (Boscaini et al. 2022). In randomized controlled trials, four standard CR care plans have been shown to be effective in reducing the health risks associated with obesity in adults. Alternate‐day fasting (ADF) is particularly effective among these therapeutic plans in reducing weight, body mass index (BMI), waist circumference, fat mass, and insulin resistance. Time‐restricted eating (TRE) appears to be more effective at reducing fasting glucose, and short‐term fasting (STF) may lead to a significant loss of lean mass.

On the other hand, more studies are needed to represent the long‐term outcome of these different CR therapeutic plans on particular populations, for example, overweight, obese, diabetic, or suffering from polycystic ovary syndrome. Each CR care plan should also undergo a thorough investigation of the various concomitant metabolic benefits, potential adverse effects, and long‐term sustainability of the measured weight loss (Huang et al. 2024). Healthy Subjects with High Caloric Restriction have shown improvements in cardiovascular and metabolic disease risk factors, including a reduction in visceral adipose tissue mass, less ectopic fat storage, a decrease in hypertension, and changes in lipid profile (Most et al. 2018). One such search program involved an individual who participated in a weight‐reducing program with calorie restriction lasting 13 weeks, resulting in approximately a 10% weight reduction. Higher weight reduction, on the other hand, was accompanied by a more substantial drop in blood glucose levels and heart rate, and by greater gut microbiota diversity. From the above discussion, it appears that gut bacterial composition, body composition, and sex affect the efficacy of the present weight loss program, thereby underscoring the need for personalized nutritional approaches (Dhakal et al. 2020). Intermittent fasting and caloric restriction have been shown to favorably affect metabolic markers and gut microbiota composition (Anton et al. 2018). However, dropout rates in RCTs are high, and variability in fasting protocols makes generalization difficult (Huang et al. 2024).

#### 4.1.6 Ketogenic Diet

This epidemic has resulted in a significant increase in the prevalence of various comorbid conditions like diabetes, hypertension, and cancer. Therefore, effective measures for managing obesity, including dietary approaches, are essential. The primary challenge is to find a diet that promotes beneficial weight loss, causes minimal adverse effects, and maintains overall health. The ketogenic diet, initially used as a supplement to epilepsy treatment, has recently emerged as a potential candidate for weight management (Drabińska et al. 2021). KD implies a high‐fat, moderate‐protein, and low‐carbohydrate intake, with an estimated average distribution of macronutrients of 70% fat, 20% protein, and 10% carbohydrates. This shift in composition facilitates the formation of ketone bodies, which have become the primary energy source, replacing glucose (Daley et al. 2023).

Humans possess metabolic flexibility that enables them to utilize ketones as an energy source, particularly when carbohydrate availability is limited. Low insulin levels enable ketogenesis, which initiates fat mass loss, conserves protein stores, and promotes insulin sensitivity and ketone metabolism. Nutritional ketosis has been shown to improve metabolic and inflammatory markers, including lipids, HbA1c, CRP, fasting insulin, and glucose, thereby aiding in weight control (Gershuni et al. 2018). The short‐term ketogenic diet may help manage hunger and increase fat oxidative metabolism, promoting weight loss (Paoli 2014). Nonetheless, long‐term maintenance of a ketogenic diet has its challenges, and the potential adverse effects, such as a rise in heart disease, fatty liver, and insulin resistance, should be taken into account. Further studies should investigate the effectiveness of ketogenic diets in promoting weight loss over various durations and in different populations (Zaman and Ahammed 2024). The ketogenic diet has been linked with short‐term improvements in fat oxidation, glycemic control, and appetite regulation (Gershuni et al. 2018). Nonetheless, concerns exist regarding cardiovascular risk, fatty liver, and adherence over time (Zaman and Ahammed 2024).

#### 4.1.7 High‐Fiber Diet

Dissolvable fibers are partially digested in an aqueous environment. They are therefore termed “soluble” fibers, while those that cannot be digested and reach the colon to undergo fermentation by gut bacteria are classified as insoluble fibers. Sources of dietary fiber include cereals, legumes, fruits, and vegetables (Williams et al. 2019). A diet rich in fiber confers numerous health benefits, including promoting regular bowel function, preserving intestinal epithelial barrier integrity, lowering serum cholesterol levels, modulating appetite, and reducing the risk of both intestinal inflammation and colorectal cancer (Akbar and Shreenath 2020). Studies investigating the impact of high‐fiber diets in obese individuals have demonstrated supportive effects, including reduced body weight gain and enhanced satiety. Dietary fibers also exhibit the potential to modulate gut microbiota composition, consistently increasing lactic acid bacteria, such as Ruminococcus, *E. rectale* , and Roseburia, while decreasing Clostridium and Enterococcus species. Furthermore, increased fiber intake promotes the proliferation of fiber‐fermenting bacteria, leading to the production of short‐chain fatty acids (SCFAs), including acetate, propionate, and butyrate. SCFAs exert diverse beneficial health effects, including improved immunity against pathogens, maintenance of blood–brain barrier integrity, provision of energy substrates, and regulation of critical intestinal functions, such as satiety hormone production, thereby contributing to appetite control and decreased energy intake (Tomova et al. 2019). High‐fiber diets improve gut microbial diversity and SCFA production, with positive impacts on satiety and immune modulation (Tomova et al. 2019), but clinical outcomes on long‐term weight maintenance are inconsistent.

### 4.2 Exercise Intervention

Thus, dietary interventions and exercise methods for controlling body weight, both of which affect gut microbiota composition, are well documented. Food intake directly impacts gut microbes by serving as their substrate for growth. Exercise causes greater uncertainty in microbial changes, depending on duration and intensity. This indicates that the diet has a greater impact on gut microbiota than exercise does (Lai et al. 2018). Exercise has different effects on gut microbiota depending on its type and intensity. Whereas voluntary wheel running (VWR) has been shown to ameliorate colonic inflammation in various mouse models of colitis, forced treadmill running (FTR) has been shown to exacerbate colonic inflammation. The differing effects of exercise likely correspond to different changes in the intestinal microbiome observed in feces and cecum samples, despite their different community structures (Allen et al. 2015).

**FIGURE 7:**
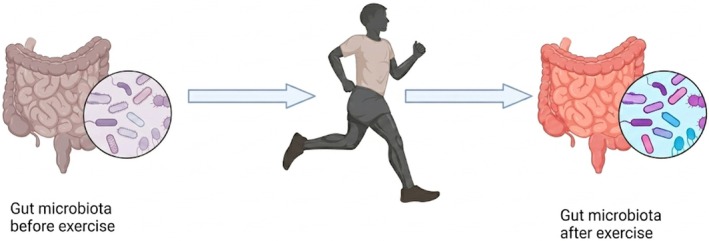
Exercise impact on gut microbial health.

A study using rats found that wheel‐running exercise resulted in a significantly different microbiota composition compared with that in seated controls. Concerning butyrate concentration in cecal samples, the exercised rats also showed an increase in n‐butyrate, which could be one of the mechanisms by which exercise may beneficially affect gastrointestinal disorders (Matsumoto et al. 2008). The gut microbiota plays a significant role in human metabolism, immune response, and development. Athletes enhance metabolic pathways (including amino acid, antibiotic biosynthesis, and carbohydrate metabolism) and fecal metabolites (SCFAs: acetate, propionate, and butyrate) associated with improved muscle turnover and health compared with sedentary individuals (Barton et al. 2018). Recently, an association was established between an organism's endurance performance and the abundance of *Bacteroides uniformis* , a producer of SCFAs, as shown in Figure 7. Targeting such an organism through dietary intervention has increased the endurance in humans and mice (Morita et al. 2023).

### 4.3 Surgical Interventions

Surgical interventions for severe obesity primarily focus on either reducing food consumption or limiting nutrient absorption (O'Brien et al. 2019). The Roux‐en‐Y gastric bypass (RYGB) is a well‐established procedure for treating morbid obesity, resulting in weight loss and improvements in metabolic and inflammatory parameters. After RYGB, augmentation of the gut microbiota, with a significant increase in Proteobacteria (37%), can be observed. The association between gut microbiota composition and white fat (WAT) gene expression is also increased. Following RYGB, 14 discriminant bacterial genera (7 dominant, 7 subdominant) and 202 WAT genes were observed to change (Kong et al. 2013). The studies suggest that the longer the time interval post‐RYGB, the greater the risk of weight regain, with younger age an important predictor even after adjusting for time since surgery. Further prospective studies will be needed to identify the prevalence, variables, and mechanisms of weight regain after RYGB (Shantavasinkul et al. 2016).

Experimental studies have shown that Sarcina abundance correlates very well with post‐surgical BMI. Outcome measures of successful weight loss are linked with increased abundance of three phyla: Firmicutes, Proteobacteria, and Bacteroidetes (Gutiérrez‐Repiso et al. 2019). On the contrary, Firmicutes are more abundant, and Bacteroidetes are less abundant in obese mice than in lean mice, irrespective of the genetic background, which may suggest that changes in the balancing of gut bacteria due to obesity can be managed for therapeutic purposes in weight management (Ley et al. 2005). Overall, bariatric surgery increases alpha diversity and induces a significant shift in beta diversity. Therefore, such differences in composition and function persist for up to 6 months post‐surgery. Correlations between microbiome changes, decreased inflammatory markers, increased bile acids, and products of choline metabolism suggest that the microbiome partially mediates metabolic improvements in the first year (Shen et al. 2019). Bariatric surgery consistently results in significant weight reduction and metabolic improvements, partly mediated by shifts in gut microbiota (O'Brien et al. 2019). However, weight regain can occur over time, and applicability is limited to severe obesity with surgical risks (Shantavasinkul et al. 2016).

### 4.4 Pharmacological Interventions

#### 4.4.1 Fecal Microbiota Transplantation (FMT)

Metabolic health modulation has redundant mechanisms in organisms, suggesting possible therapeutic interventions from homo fecal microbiota transplants (FMTs). FMT has been proven to be a curative procedure for recurring Clostridioides difficile infection (CDI) (Baunwall et al. 2020) and has been investigated for inflammatory bowel disease (Imdad et al. 2023). However, the outcome of studies on the application of FMT on weight control and obesity in humans produced contradictory outcomes, such as no measurable clinical impact (Yu et al. 2020) and positive results on secondary outcomes, including body composition, well‐being, and gut microbiome composition. In a randomized clinical trial involving adolescents suffering from obesity, the FMT administered did not significantly affect weight loss, though a reduction in abdominal adiposity occurred (Leong et al. 2020). On the contrary, a reported research indicated that single‐dose oral FMT, when added with a back‐to‐back low fermentable fiber supply, evinced improvement in insulin sensitivity, unlike findings seen in offering the severely obese and the subjects with metabolic syndrome (Mocanu et al. 2021).

Autologous FMT is the collection of one's feces during weight loss, and its administration during weight regain, which may prevent weight and glycemic control correlated with specific microbiome profiles (Rinott, Youngster, Meir, et al. 2021). Likewise, high polyphenol, green plant‐based, or Mankai diets may optimize an individual's microbiome for autologous FMT (Rinott, Youngster, Yaskolka Meir, et al. 2021). Application of live or pasteurized *Akkermansia muciniphila* cultivated in synthetic medium is considered safe in humans and may reduce fat mass development, improve insulin resistance, and mitigate climate change by preventing low‐grade inflammation through improved gut barrier integrity (Plovier et al. 2017). In a clinical trial, oral supplementation of 1010 pasteurized *A. muciniphila* was safe and well‐tolerated when administered daily for 3 months, significantly improving insulin sensitivity and lowering insulinemia, with minimal changes in body weight and hip circumference from baseline. These studies imply that pasteurized *A. muciniphila* is far better than the live form (Depommier et al. 2019). Clinical evidence on FMT for obesity is inconclusive. Some RCTs demonstrate improved insulin sensitivity and reduced visceral adiposity (Leong et al. 2020), while others report no significant impact on weight (Yu et al. 2020). Donor variability, safety, and long‐term microbiota engraftment remain major challenges (Rinott, Youngster, Meir, et al. 2021).

#### 4.4.2 Antibiotics and Antimicrobials

In this study, antibiotic treatment was shown to impair gut microbiota function and reduce weight gain in those mice. Probiotic administration, on the other hand, with *Lactobacillus plantarum* or *Lactobacillus rhamnosus* GG counteracts this effect by shifting gut bacterial equilibrium and lessening the harm of antibiotics (Miao et al. 2020). The disruption of gut microbiota composition by early antibiotic exposure can alter its metabolic activity, leading to either increased weight gain or growth inhibition in the host (Azad et al. 2014). In an experimental study, antibiotic treatment of obesity‐prone C57BL/6J mice fed a high‐fat diet (HFD) altered intestinal microbiota, reduced tissue inflammation, improved insulin resistance, and enhanced glucose metabolism. Physiological changes observed were also seen after transferring gut microbiota from antibiotic‐treated donors to germ‐free or germ‐depleted mice, correlating with the changes in serum bile acids. In contrast, antibiotic intervention in HFD‐fed obesity‐resistant 129S1 and obesity‐prone 129S6 mice failed to improve metabolism, despite altering the microbiota and bile acids, suggesting that the metabolic benefits of antibiotics depend on the host's genetic background (Fujisaka et al. 2016). Variations in the genetic code related to body mass index (BMI) and waist‐to‐hip ratio (WHR) in adults would affect growth patterns and overall and abdominal fat deposition from early childhood (Vogelezang et al. 2015). Cesarean section and antibiotics during pregnancy may prevent the normal transmission of maternal and infant microbiota, thereby contributing to faulty colonization of the infant gut by microbes and increasing susceptibility to obesity later in life (Mueller et al. 2015).

## 5 Conclusion

The gut microbiome contributes to obesity through multiple mechanisms, including altered energy harvesting, SCFA signaling, regulation of the gut–brain axis, bile acid metabolism, and impaired barrier integrity. Interventions such as probiotics, prebiotics, synbiotics, intermittent fasting, ketogenic and high‐fiber diets, pharmacological agents, FMT, and bariatric surgery have shown potential to beneficially modulate the gut microbiota and improve metabolic outcomes. Clinically, these findings underscore the promise of microbiome‐targeted strategies as adjuncts in the prevention and treatment of obesity. However, major research gaps remain, including the limited availability of large‐scale longitudinal trials, variability in individual microbial responses, and uncertainty about the long‐term safety and sustainability of these approaches. Future research should prioritize standardized protocols, precision nutrition approaches tailored to microbial signatures, and integrative strategies that combine diet, exercise, pharmacological, and microbial interventions. Advancements in multi‐omics and personalized medicine hold promise for developing more effective and sustainable microbiome‐based therapies.

## Author Contributions

**Noman Ali:** conceptualization, investigation, validation, software, resources. **Ayesha Rehman:** conceptualization, visualization, formal analysis. **Abdikhaliq Mursal Yusuf:** funding acquisition, formal analysis, software, visualization, writing – review and editing. **Azeem Mushtaq:** conceptualization, investigation, methodology, writing – review and editing, software. **Muhammad Rizwan Tariq:** writing – original draft, supervision, resources, project administration. **Shinawar Waseem Ali:** investigation, writing – original draft, visualization, project administration, supervision. **Waseem Safdar:** methodology, formal analysis, project administration, data curation, writing – original draft.

## Conflicts of Interest

The authors declare no conflicts of interest.

## Data Availability

The data that support the findings of this study are available from the corresponding author upon reasonable request.
